# A Machine Learning Trauma Triage Model for Critical Care Transport

**DOI:** 10.1001/jamanetworkopen.2025.9639

**Published:** 2025-06-09

**Authors:** Aaron C. Weidman, Salim Malakouti, David D. Salcido, Chase Zikmund, Ravi Patel, Leonard S. Weiss, Michael R. Pinsky, Gilles Clermont, Jonathan Elmer, Ronald K. Poropatich, Joshua B. Brown, Francis X. Guyette

**Affiliations:** 1 Department of Emergency Medicine, University of Pittsburgh School of Medicine, Pittsburgh, Pennsylvania; 2 NOMA AI Inc, Pittsburgh, Pennsylvania; 3 Department of Critical Care Medicine, University of Pittsburgh School of Medicine, Pittsburgh, Pennsylvania; 4 Center for Military Medicine Research, University of Pittsburgh School of Medicine, Pittsburgh, Pennsylvania; 5 Department of Surgery, University of Pittsburgh School of Medicine, Pittsburgh, Pennsylvania

## Abstract

**Importance:**

Under austere prehospital conditions, rapid classification of injured patients for intervention or transport is essential for providing lifesaving care. Discerning which patients need care most urgently further allows for optimal allocation of limited resources. These triage processes are hindered by the limited diagnostic resources and modalities available in the prehospital environment.

**Objective:**

To develop a triage model for prehospital use in patients with traumatic injury supported by machine learning (ML) analysis of continuous physiological waveform signals and derived patterns of vital signs.

**Design, Setting, and Participants:**

This retrospective cohort study used data from January 1, 2018, to November 18, 2021, from critically ill patients with trauma transported by a large critical care air transport system serving Pennsylvania and surrounding states. Patients were included if classified as a trauma case by treating prehospital clinicians during a scene run by the transport service. Data were analyzed from May to November of 2024.

**Exposures:**

Metrics derived from physiological waveform signal and vital sign patterns during the first 15 minutes following initiation of patient care and transport.

**Main Outcomes and Measures:**

Administration of a lifesaving intervention (LSI) occurring within a 2-minute epoch during patient care. An ensemble ML approach was applied to predict LSI occurrence from physiological features recorded in the 2-minute epoch immediately preceding the LSI epoch.

**Results:**

A total of 2809 participants were included in the analysis (mean [SD] age, 47.7 [19.5] years; 1981 [70.5%] men). These participants had 15 088 two-minute epochs that yielded physiological data recording, of which 910 (6.0%) included an LSI. Good model performance was observed for predicting overall LSI, with an area under the receiver operating characteristics curve of 0.810 (95% CI, 0.782-0.842); sensitivity, 0.268 (95% CI, 0.193-0.357); positive predictive value, 0.301 (95% CI, 0.228-0.356); positive likelihood ratio, 6.793 (95% CI, 4.887-8.795); specificity, 0.960 (95% CI, 0.947-0.972); negative predictive value, 0.953 (95% CI, 0.943-0.964); and negative likelihood ratio, 0.763 (95% CI, 0.680-0.837). Performance was equivalent or better when predicting several LSI subcategories (eg, airway intervention, blood transfusion, vasopressor medication), when using physiological features captured up to 15 minutes prior to LSI administration, when predicting only the first LSI occurrence for each patient, and across mechanism of injury.

**Conclusions and Relevance:**

In this cohort study of critically ill patients with trauma in the prehospital setting, an ML-based triage model using physiological features provided accurate predictions of lifesaving intervention delivery to single patients. Modeling approaches could be deployed in the field to help streamline and augment prehospital triage.

## Key Points

**Question:**

Can machine learning analysis of patient physiological signals predict the need for prehospital lifesaving intervention?

**Findings:**

In this cohort study of 2809 patients with trauma and critical illness, continuous physiologic waveform signals and derived vital sign patterns were fed into an ensemble machine learning classifier to predict administration of prehospital lifesaving interventions within 2-minute treatment epochs (eg, airway intervention, blood transfusion). The model achieved good performance, which was maintained using physiological features captured up to 15 minutes prior to intervention.

**Meaning:**

These findings suggest that modeling approaches have the potential to streamline and augment prehospital trauma triage.

## Teaser

This cohort study describes the development of a machine learning model to identify single-patient administration of lifesaving interventions from a brief epoch of physiological waveform data among critically ill patients with trauma in the prehospital setting.

## Introduction

Advances in civilian casualty care have the potential to reduce mortality from trauma. Most trauma deaths are related to airway compromise or prehospital hemorrhage^1,2^; as many as one-quarter are preventable with early recognition and delivery of lifesaving interventions (LSI).^3^ However, prehospital medics determining the need for LSI face a daunting task: having to rapidly assess multiple casualties and make critical decisions about required care and resource utilization. Medics must complete these tasks with limited skill and experience relative to in-hospital clinicians, minimal time and resources, and a lack of advanced diagnostic tools.^4^ Existing triage guidelines meant to streamline prehospital decision-making are often unreliable, due in part to inclusion of cues that do not always reliably identify patients who require immediate LSI (eg, vital signs, anatomical injury patterns).^5,6,7^

Machine learning (ML) analyses of patient physiological features may augment prehospital decision-making. Physiological monitoring can capture complex high-dimensional waveforms via noninvasive technologies in prehospital settings. Waveform data are superior to traditionally recorded vital signs in estimating mortality risk, LSI need, and triage,^6,8,9,10^ due in part to their ability to presage clinical decompensation. Heart rate variability and complexity are associated with LSI need and hemorrhage risk.^11,12,13,14,15,16,17^ Photoplethysmography waveforms outperform enroute vital sign data for similar tasks.^18,19,20,21^ ML allows rapid and automated detection of subtle patterns from complex physiological signals, making it a powerful tool for constructing prediction models based on waveform and derived vital sign data.^22,23^

We developed an ML model capable of identifying single-patient LSI administration from a brief epoch of physiological waveform data. We examined how this model performs using standard ML metrics and per-patient overtriage and undertriage rates. We hypothesized that our model would yield performance on par with reported performance in existing triage models while yielding overtriage and undertriage rates similar to goals set in the national triage guidelines.^24,25^

## Methods

### Overview

For this cohort study, we obtained patient data from a medical transport organization (STAT MedEvac) that serves approximately 13 000 patients annually. The University of Pittsburgh Institutional Review Board approved this retrospective cohort study under a waiver of informed consent given that it involved observational research conducted on existing medical records. We followed the Strengthening the Reporting of Observational Studies in Epidemiology (STROBE) and the Transparent Reporting of a Multivariable Prediction Model for Individual Prognosis or Diagnosis (TRIPOD-AI) reporting guidelines. Race and ethnicity are reported for sample description purposes, but demographic information was not used in ML modeling.

**Figure 1.**
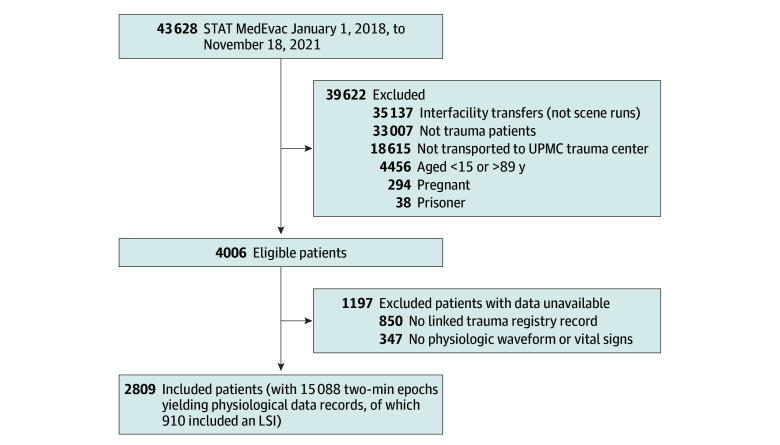
Patient Inclusion Flow Diagram Patients could be deemed ineligible due to more than 1 criterion. As a result, the subtotal of ineligible excluded patients exceeds the total of 39 622. We observed a similar demographic profile between included patients and those excluded due to unavailable data (eTable 1 in Supplement 1). LSI indicates lifesaving intervention; UPMC, University of Pittsburgh Medical Center.

Emergency 911 calls are assigned to medical air transport based on state protocols regarding severity, time sensitivity, and distance from the nearest appropriate facility. Medical air transport scene runs spanning January 1, 2018, to November 18, 2021, were eligible if the case was classified as a trauma by treating prehospital clinicians; if the case involved direct transport of patients from the scene of patient injury to 1 of 4 adult trauma centers in the University of Pittsburgh Medical Center hospital system (patients transferred between facilities were ineligible); if patients were aged 15 to 89 years; and if patients were suspected to be neither pregnant nor a prisoner. Of eligible patients (n = 4006), we excluded those with incomplete data (ie, no available monitor file containing continuous physiological waveform and vital sign patterns or no linked record in our in-hospital trauma registry) (Figure 1). We observed a similar demographic profile between our included sample (n = 2809) and eligible patients excluded due to incomplete data (n = 1197) (eTable 1 in Supplement 1).

A mean (SD) of 22.6 (8.5) minutes elapsed between 911 dispatch and medical air transport crew arrival at the patient’s location; we do not have patient data from this period. Once the medical air transport crew arrived, they began to record physiological and waveform data, which was inputted to an electronic patient care reporting system (emsCharts; ZOLL Data Systems). We extracted data from the reporting software for analysis, including transport timing and timestamped vital signs and procedures. We downloaded continuous physiological waveforms and 30-second mean vital sign patterns from the corresponding patient monitor (X-series; ZOLL Medical). Available sensor modalities and derivatives included electrocardiography and heart rate; finger plethysmography and oxygen saturation by pulse oximeter; airway capnography and end-tidal carbon dioxide; and both invasive blood pressure (IBP; taken via arterial catheterization) and non-IBP (taken using a cuff). We analyzed data collected during the first 15 minutes following the arrival of the medics at the patient to identify patients who most urgently required LSI.

### Physiologic Feature Creation

We parsed each patient’s physiological waveform data in contiguous nonoverlapping 2-minute epochs within the initial 15-minute period following prehospital clinician arrival. We derived statistical representations capturing patient physiological condition during each 2-minute epoch. Two-minute epochs allowed for time-specific predictions of patient LSI within the initial 15-minute care window while providing sufficient duration to calculate statistical features based on physiological waveform data captured (eg, heart rate variability).^26^ A full list of the 2175 unique statistical features initially computed is available on the Open Science Framework.^27^

### LSI Outcome Extraction

We used prehospital emergency medical record and trauma registry data to identify the occurrence of specific treatments constituting LSI during the 15-minute analysis period. Following previously proposed definitions,^28^ we determined inclusion of each treatment via iterative discussion among several authors with clinical prehospital knowledge (L.S.W., J.B.B., and F.X.G.); these authors were blinded to the effect of their decisions on predictive modeling. We split LSI into 6 categories: airway interventions (eg, endotracheal intubation), bleeding control (eg, pelvic binder), blood transfusion (eg, whole blood), cardiovascular interventions (eg, cardiopulmonary resuscitation), thoracic interventions (eg, needle decompression), and vasopressor medication (eg, epinephrine).

**Figure 2.**
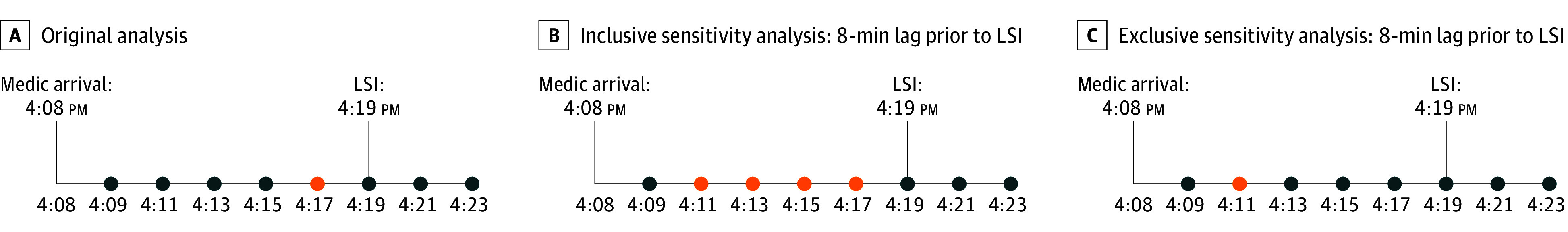
Schematic for Lifesaving Intervention (LSI) Prediction From 2-Minute Treatment Epochs Hypothetical diagram depicting schematic for predicting LSI from physiological features derived from preceding 2-minute treatment epochs. Each dot marks the beginning of a 2-minute epoch. Patient encounter begins at 4:08 pm; LSI was administered at 4:19 pm. Dark blue circles indicate epochs that would not be used to predict the LSI occurring at 4:19 pm. A, The orange circle indicates a 2-minute epoch that would be used to predict the LSI at 4:19 pm, given that an LSI occurred 1 to 2 minutes following this epoch. B, The orange circles indicate 2-minute epochs that would be used to predict the LSI at 4:19 pm, given that an LSI occurred 8 minutes or less following the start of each of these epochs. C, The orange circle indicates a 2-minute epoch that would be used to predict the LSI at 4:19 pm, given that an LSI occurred exactly 8 minutes following the start of this epoch.

We matched timestamped LSIs to the immediately preceding 2-minute epoch and associated physiological feature set. Each epoch was linked to a binary positive or negative outcome based on whether an LSI immediately followed (1 indicates LSI; 0, no LSI). Patients could have as many as 8 epochs across the 15-minute analytic period, with a final 1-minute epoch for some patients (Figure 2). This briefer epoch could only be used as an outcome based on whether it involved LSI; physiological waveform data collected during this epoch were not used for prediction. The eFigure in Supplement 1 presents examples of patient timelines.

### Statistical Analysis

#### Machine Learning

We used ML to predict prehospital LSI administration during each 2-minute epoch from physiological features recorded during the immediately preceding epoch (Figure 2). We deployed histogram gradient boosting (HGB), an ensemble method, via Scikit-learn in Python (Python Software Foundation).^29^ HGB involves fitting a series of decision trees to the data; each successive decision tree learns from errors of the prior tree, with the patient receiving a binary classification based on the final tree. Models were trained using all LSIs administered across all patients, including patients who received multiple LSIs; for these patients, each LSI was predicted independently via physiological features recorded during the preceding 2-minute epoch, regardless of the order in which specific types of LSIs were administered. Separate models were run for each LSI category.

Model performance was evaluated via the area under the receiver operating characteristics curve (AUROC), which compares model sensitivity (ie, correctly detecting true LSI cases) with false-positive rates. We also report several classification metrics, including sensitivity, positive predictive value, positive likelihood ratio, specificity, negative predictive value, and negative likelihood ratio. We calculated these metrics by setting the model threshold such that the percentage of predicted LSI cases roughly equaled the observed epoch-wide LSI rate. Model performance was visualized graphically using ggplot2 in R.^30^

To interface with prior prehospital trauma triage work, we also calculated per-patient undertriage and overtriage rates based on model predictions. Undertriage is defined as the share of patients predicted not to need an LSI across all 2-minute epochs who did receive at least one LSI (ie, 1 – sensitivity; false-negative rate). Overtriage is defined as the share of patients predicted to need an LSI during at least one 2-minute epoch who did not receive any LSI (ie, 1 – specificity; false-positive rate). Following field triage guides,^25^ we set the overtriage rate to be 35% or less and observed how close the undertriage rate came to the national goal of 5% or less. Using the resultant prediction threshold, we also reported each of the previously mentioned classification metrics at a per-patient level.

We performed a grid search for optimal hyperparameter settings in HGB ML models; 100 maximum iterations, a maximum tree depth of 3, and a learning rate of 0.1 were chosen based on a high AUROC (eTable 2 in Supplement 1). We used 5-fold cross-validation with an 80% training and 20% evaluation split, stratified by patient to prevent overfitting (ie, all of a patient’s 2-minute epochs appeared in either the training or evaluation data). We used 20 bootstrapped resamples, each with a unique random seed to ensure robustness. Combined with 5-fold cross-validation, this approach yielded 100 unique model runs and resultant 95% CIs for each model metric. We reran our models predicting several extremely rare LSI categories while using an oversampling technique to increase representation of these LSIs (eAppendix in Supplement 1).

For sensitivity analysis, we tested whether LSI prediction would be maintained when using earlier recorded physiological features (Figure 2). We used as predictors 2-minute epochs that occurred increasingly antecedent to each administered LSI as predictors, up to 15 minutes prior. In an inclusive version, we used all 2-minute windows up to and including the most antecedent window; an 8-minute inclusive window implies that each epoch between 4:11 and 4:18 pm would be used to predict whether an LSI occurred at 4:19 pm. In an exclusive version, we included only the most antecedent 2-minute window; an 8-minute exclusive window implies that the epoch at 4:11 to 4:12 pm would be used to predict whether an LSI occurred at 4:19 pm but that epochs occurring from 4:13 to 4:18 pm would not be used. We further tested whether predictions would be maintained when (1) restricting the model to predicting the first LSI per person (subsequent 2-minute epochs were discarded, yielding 13 495 epochs [89.4%] of the original 15 088; 616 LSI events for an LSI rate of 4.56%); (2) only examining patients with blunt trauma (too few patients had penetrating trauma to support a subgroup analysis); and (3) using median imputation to fill missing values.

Data were analyzed from May to November of 2024. Two-sided *P* < .05 indicated statistical significance.

## Results

### Overview

A total of 2809 patients were included in the analysis (mean [SD] age, 47.7 [19.5] years); of these, 1981 [70.5%] were men, 775 [27.6%] were women, and 53 [1.9%] other. In terms of race and ethnicity, 115 (4.1%) patients were Black, 55 (2.0%) were Hispanic, 2545 (90.6%) were White, 12 (0.4%) were other, and 82 (2.9%) were unknown or missing. Among those with mechanism of injury identified, 2535 patients (90.3%) had blunt trauma and 202 (7.2%) had penetrating trauma. LSIs occurred in 616 patients (21.9%) among included patients, statistically equivalent to the rate among excluded patients (272 of 1197 [22.7%]; *z* = 0.554; *P* = .58). Included patients who received at least one LSI received a mean (SD) of 1.48 (0.79) LSIs (409 [66.4%] received only 1), significantly less than among excluded patients who received at least one (mean [SD], 2.07 [1.89] LSI; 143 [52.6%] received only 1; *t* = 6.56; *P* < .001) (Table 1). Patients had a mean (SD) of 5.38 (0.73) 2-minute epochs (range, 3-8; total, 15 088); patients could have had fewer than the maximum of 8 epochs due to missing physiological waveform data during 1 or more epochs or termination of care by air transport medics prior to 15 minutes having elapsed. A total of 616 patients (21.9%) received an LSI within 15 minutes, whereas only an additional 161 (5.7%) received an LSI between 16 and 30 minutes.

**Table 1.** Rates for Procedures Classified as LSIs

LSI	No. (%) of LSIs		
	Excluded patients (n = 1197)	Included patients (n = 2809)	2-min Epochs (n = 15 088)
Airway interventions	165 (13.8)	366 (13.0)	532 (3.5)
Bag-valve-mask	95 (7.9)	171 (6.1)	206 (1.4)
Cricothyrotomy	0	0	0
Direct laryngoscopy	10 (0.8)	19 (0.7)	20 (0.1)
Endotracheal intubation	134 (11.2)	299 (10.6)	313 (2.1)
i-Gel supraglottic	0	3 (0.1)	3 (0.02)
King Airway LTS-D/Combitube	2 (0.2)	1 (0.04)	1 (0.01)
NIPPV	0	0	0
Oral/nasal pharyngeal airway	18 (1.5)	28 (1.0)	28 (0.2)
Ventilator	32 (2.7)	65 (2.3)	67 (0.4)
Video laryngoscopy	128 (10.7)	283 (10.1)	295 (2.0)
Bleeding control	100 (8.4)	194 (6.9)	200 (1.3)
Pelvic binder	37 (3.1)	47 (1.7)	48 (0.3)
Tourniquet^a^	0	2 (0.1)	2 (0.01)
Tranexamic acid	27 (2.3)	77 (2.7)	77 (0.5)
Wound control (eg, pressure; bandaging, clamp)	29 (2.4)	78 (2.8)	79 (0.5)
Blood transfusion	28 (2.3)	94 (3.3)	99 (0.7)
Blood components (plasma, platelets, packed red blood cells)	27 (2.3)	89 (32)	94 (0.6)
Whole blood	1 (0.1)	5 (0.2)	5 (0.03)
Vasopressor medication	28 (2.3)	54 (1.9)	69 (0.5)
Dopamine	0	0	0
Dobutamine	0	0	0
Epinephrine	26 (2.2)	48 (1.7)	61 (0.4)
Norepinephrine	7 (0.6)	8 (0.3)	8 (0.05)
Phenylephrine	0	0	0
Vasopressin	0	0	0
Thoracic interventions	17 (1.4)	37 (1.3)	40 (0.3)
Chest tubes	1 (0.1)	0	0
Needle decompression	17 (1.4)	37 (1.3)	40 (0.3)
Cardiovascular interventions	9 (0.8)	10 (0.4)	21 (0.1)
CPR	9 (0.8)	10 (0.4)	21 (0.1)
Mechanical CPR	0	3 (0.1)	7 (0.05)
All	272 (22.7)	616 (21.9)	910 (6.0)
None	925 (77.3)	2193 (78.1)	14 178 (94.0)

Abbreviations: CPR, cardiopulmonary resuscitation; LSI, lifesaving intervention; NIPPV, nasal intermittent positive pressure ventilation.

^a^ Tourniquet administration must be accompanied by a blood component infusion, whole blood infusion, or cardiac defibrillation to be classified as an LSI.

### Feature Selection

Due to the complex nature of data collection and the statistical transformations, we observed high rates of missingness among the initial 2175 physiological features. We excluded 550 features with near-complete missingness, including all IBP features (n = 450) and several non-IBP features (n = 75). Features reflecting fractional inspiratory carbon dioxide (n = 25) were also excluded; although routinely collected by patient monitors along with end-tidal carbon dioxide, fractional inspiratory carbon dioxide reflects ambient levels of carbon dioxide in the atmosphere, not patient physiological condition.

We retained 1625 features for our model; complete data were observed for only 17 (0.1%) 2-minute epochs across patients, requiring us to account for 2 missingness types. The first type involved calculations that were not possible due to signal noise or abnormal data readings (2.2% of the more than 24.5 million unique values across 15 088 two-minute epochs multiplied by 1625 physiologic features); these were treated with feature-wise median imputation or zeroes were imputed when a median could not be computed for a feature. The second type involved absent physiological waveform data for a given patient during a specific 2-minute epoch (16.3% of values). Given the potential utility of this second type of missing data in identifying patient deterioration, we explicitly represented it as a feature, allowing it to contribute organically to model prediction.

### ML Modeling

#### Per-Epoch Analysis

**Figure 3.**
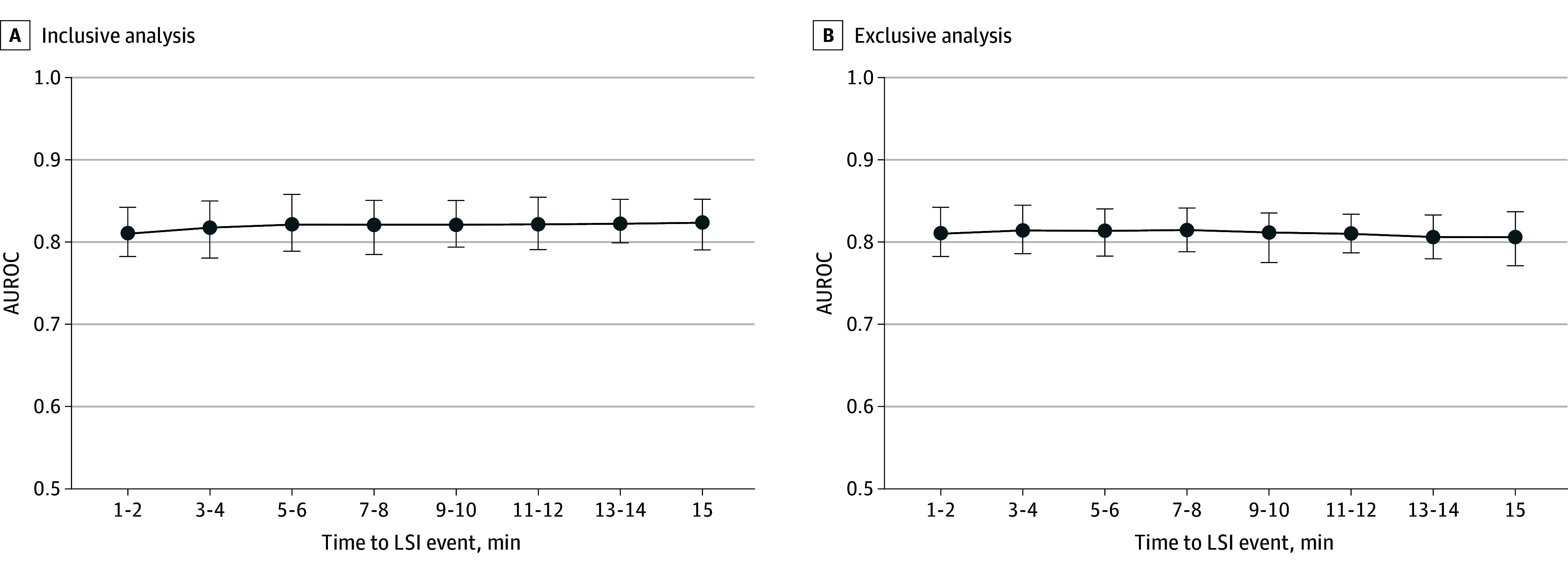
Machine Learning Results Predicting Lifesaving Intervention (LSI) From Physiological Features Time to LSI event of 1 to 2 minutes corresponds to the primary analysis reported; results are identical across inclusive and exclusive panels. All other data points correspond to the sensitivity analysis; results diverge across inclusive and exclusive panels. A, All 2-minute epochs beginning at or after the specified time to LSI event are used to predict LSI administration during the target epoch. B, Only the 2-minute epoch beginning at the specified time to LSI event is used to predict LSI administration during the target epoch. Vertical bars represent 95% CIs. AUROC indicates area under the receiver operating characteristics curve.

We examined whether LSI could be predicted from proximal physiological features (Table 2 and Figure 3). We observed good performance, with an AUROC of 0.810 (95% CI, 0.782-0.842); sensitivity, 0.268 (95% CI, 0.193-0.357); positive predictive value, 0.301 (95% CI, 0.228-0.356); positive likelihood ratio, 6.793 (95% CI, 4.887-8.795); specificity, 0.960 (95% CI, 0.947-0.972); negative predictive value, 0.953 (95% CI, 0.943-0.964); and negative likelihood ratio, 0.763 (95% CI, 0.680-0.837). Compared with model performance for overall LSI, model performance for airway interventions was stronger, with an AUROC of 0.910 (95% CI, 0.888-0.932). Model performance for blood transfusions (AUROC, 0.784; 95% CI, 0.688-0.872) and vasopressor medications (AUROC, 0.816; 95% CI, 0.652-0.916) was equivalent; model performance for bleeding control (AUROC, 0.580; 95% CI, 0.486-0.658), thoracic interventions (AUROC, 0.675; 95% CI, 0.478-0.828), and cardiovascular interventions (AUROC, 0.650; 95% CI, 0.222-0.992) was weaker.

**Table 2.** Machine Learning Results Predicting LSI and Each Specific Category

LSI administered^a^	Per-epoch rate	Metric (95% CI)						
		AUROC	Sensitivity	Positive predictive value	Positive likelihood ratio	Specificity	Negative predictive value	Negative likelihood ratio
All	0.060	0.810 (0.782-0.842)	0.268 (0.193-0.357)	0.301 (0.228-0.356)	6.793 (4.887-8.795)	0.960 (0.947-0.972)	0.953 (0.943-0.964)	0.763 (0.680-0.837)
Airway intervention	0.0352	0.910 (0.888-0.932)	0.277 (0.178-0.378)	0.259 (0.193-0.335)	9.726 (6.533-13.891)	0.971 (0.960-0.980)	0.973 (0.967-0.979)	0.745 (0.640-0.841)
Bleeding control	0.0133	0.580 (0.486-0.658)	0.018 (0.000-0.057)	0.019 (0.000-0.066)	1.475 (0.000-5.481)	0.987 (0.980-0.993)	0.987 (0.983-0.991)	0.995 (0.955-1.020)
Blood transfusion	0.0066	0.784 (0.688-0.872)	0.040 (0.000-0.140)	0.042 (0.000-0.188)	7.056 (0.000-29.719)	0.994 (0.989-0.998)	0.994 (0.991-0.996)	0.966 (0.867-1.009)
Vasopressor medication	0.0046	0.816 (0.652-0.916)	0.064 (0.000-0.238)	0.057 (0.000-0.212)	14.472 (0.000-62.243)	0.996 (0.991-0.999)	0.996 (0.993-0.998)	0.940 (0.765-1.007)
Thoracic intervention	0.0027	0.675 (0.478-0.828)	0.002 (0.000-0.002)	0.002 (0.000-0.001)	0.463 (0.000-1.641)	0.997 (0.991-1.000)	0.997 (0.996-0.999)	1.001 (1.000-1.009)
Cardiovascular intervention	0.0014	0.650 (0.222-0.992)	0.031 (0.000-0.500)	0.015 (0.000-0.173)	16.445 (0.000-177.590)	0.998 (0.993-1.000)	0.999 (0.996-1.000)	0.968 (0.502-1.007)

Abbreviations: AUROC, area under the receiver operating characteristics curve; LSI, lifesaving intervention.

^a^ Includes 15 088 two-minute epochs. All metrics are calculated at the per-epoch level. The 95% CIs are calculated via empirical bootstrapping.

#### Per-Patient Analysis

We calculated per-patient classification metrics based on whether a patient was predicted to receive and/or received at least 1 LSI. Setting the per-patient overtriage rate to 34.9% (764 of 2190 patients who did not receive LSI), we observed an undertriage rate of 21.3% (131 of 616 patients who received an LSI). These results corresponded to the following classification statistics: sensitivity of 0.787, positive predictive value of 0.388, positive likelihood ratio of 2.257, specificity of 0.651, negative predictive value of 0.916, and negative likelihood ratio of 0.443. These values were computed arithmetically based on observed counts of model-predicted and actual LSI events; as a result, 95% CIs are not reported.

### Sensitivity Analyses

In our inclusive analysis, model performance remained steady, even showing a slight increase, when adding earlier 2-minute epochs as predictors (AUROCs ranged from 0.817 [95% CI, 0.780-0.850] at 4 minutes to 0.823 [95% CI, 0.790-0.852] at 15 minutes vs 0.810 at baseline) (Figure 3). In our exclusive analysis, model performance remained consistent, with AUROCs ranging from 0.814 (95% CI, 0.786-0.845) at 4 minutes to 0.806 (95% CI, 0.771-0.837) at 15-minutes. We also observed model performance equivalent to our original analyses when (1) examining each patient’s first LSI (AUROC, 0.796; 95% CI, 0.763-0.827); (2) examining patients with blunt trauma (AUROC, 0.809; 95% CI, 0.774-0.844); and (3) using median imputation to fill missing values (AUROC, 0.810; 95% CI, 0.768-0.842).

## Discussion

In this cohort study of critically ill patients with trauma, continuous physiological waveform data acquired early in prehospital care was used to accurately predict whether a single patient received an LSI within a 2-minute epoch during transport. Our model produced a 21.3% undertriage rate, higher than the national goal of 5% or less, and an overtriage rate of 34.9%, below the national goal of 35% or less.^25^ Accurate LSI prediction was maintained for select LSI categories (eg, vasopressor medication, airway intervention, and blood transfusion), up to 15 minutes prior to LSI occurrence, and when predicting only the first LSI for each patient.

Our results have implications for prehospital triage. Undertriage and overtriage rates are higher in practice than what is recommended by national guidelines,^25^ due in part to reliance on minimal objective data (vital signs), subjective assessments (anatomical injury patterns), and often nothing more than prehospital clinician intuition.^8,31,32,33^ Our model produces undertriage and overtriage rates that compare favorably with published rates from prior clinical triage models^34,35,36,37,38^ and are not too far removed from national goals.^25^ Our model also makes time-specific LSI predictions^39^ by providing a predicted probability of whether a single patient will receive an LSI at a given time during prehospital transport. In clinical contexts in which medics weigh the needs of multiple injured patients, an analogous model might conceivably help to determine when and in what order patients receive LSI. Identifying the patients with the greatest need in an automated manner could support triage decisions that often prove challenging for prehospital clinicians. Our findings are potentially useful for the field, dovetailing with a burgeoning body of work showing that prediction models can leverage prehospital triage criteria to guide triage decisions.^13,14,15,16,17,34,35,36,37,38,39^

Our model could integrate with and streamline prehospital clinician workflow by identifying a prehospital care event (LSI) that typically follows prolonged patient decompensation. Model predictions could therefore provide an early indication for treatment decisions that usually require continuous and cognitively demanding monitoring.^8^ After determining LSI need, clinicians can focus their energies elsewhere while retaining confidence that they have not omitted a critical intervention. Freeing prehospital clinicians—often medics or nurses—to concentrate on the most severe or ambiguous cases is critical given that these individuals often lack advanced skill and experience. Clinician demand is further magnified in austere prehospital environments, increasing the potential utility of model-based decision tools. Our model also uses as inputs the same physiological and vital sign data that a prehospital clinician would routinely monitor but incorporates a much greater quantity of information than a human could compute. Ease of use and transparency should help promote uptake among medics who are wary of black box technologies that complicate their workflow.

### Limitations

Our study has several limitations. First, the fact that a patient received an LSI does not necessarily mean that the intervention was indicated; future studies should examine physiological sequelae of prehospital LSI to determine whether model-based predictions of LSI made up to 15 minutes in advance reliably indicate need for LSI administration. Second, data were derived from a critical care database in which most patients were transported via air, and results may not generalize to different prehospital patient populations; future work should externally validate in populations primarily undergoing prehospital ground transport. Third, LSI timestamps were taken from the prehospital emergency medical record and therefore may be subject to recording errors; our sensitivity analysis, in which model performance was maintained up to 15 minutes prior to LSI, mitigates this concern. Fourth, we examined the first 15 minutes following medic arrival, given that most LSIs would occur during this time (indeed, 616 [21.9%] patients received an LSI within 15 minutes, whereas only an additional 161 [5.7%] received an LSI between 16 and 30 minutes); an open question is whether the predictive models developed herein generalize to later stages of emergency care (eg, interfacility transport or in hospital). Fifth, given the rarity of most specific LSIs, we did not have statistical power to examine variability across patients who received multiple LSIs in different orders. Sixth, some patients were excluded as their injury severity precluded obtaining monitor data, possibly introducing a spectrum bias.

## Conclusions

In this cohort study of critically ill patients with trauma, we presented an ML model trained to identify single-patient need for LSI during 2-minute prehospital care epochs based on immediately preceding physiological waveform features. The model maintained good performance up to 15 minutes prior to intervention and accurately predicted several specific lifesaving procedures (eg, airway intervention and blood transfusion). ML approaches could augment prehospital triage, pending further evaluation in broad emergency populations and across different phases of emergency care.

## Notes

Supplement 1. **eAppendix.** SMOTE Analyses for Very Rare LSI **eTable 1.** Demographic Profile of Included Patients vs Eligible Patients Excluded Due To Incomplete Data **eTable 2.** Grid Search Results for Histogram Gradient Boosting Models **eFigure.** Prehospital Care Timelines for 3 Patients in the Included Cohort (n = 2809)
eReference

Supplement 2.
Data Sharing Statement
