# Error in Statistical Analysis Section

**DOI:** 10.1001/jamanetworkopen.2025.25559

**Published:** 2025-07-09

In the Original Investigation titled “A Machine Learning Trauma Triage Model for Critical Care Transport,”^1^ published June 9, 2025, there was a wording error in the Statistical Analysis section. In the statement regarding undertriage, the parenthetical phrase given as “(ie, 1 – specificity; false-negative rate)” should have been “(ie, 1 – sensitivity; false-negative rate).” In the statement regarding overtriage, the parenthetical phrase given as “(ie, 1 – sensitivity; false-positive rate)” should have been “(ie, 1 – specificity; false-positive rate).” This article has been corrected.^1^
