# Supplementary material for: A Machine Learning Trauma Triage Model for Critical Care Transport
Source: JAMA Netw Open. 2025 Jun 9;8(6):e259639. doi: 10.1001/jamanetworkopen.2025.9639 (PMC12150194; doi:10.1001/jamanetworkopen.2025.9639)
Supplement: Supplement 1. — eAppendix. SMOTE Analyses for Very Rare LSI eTable 1. Demographic Profile of Included Patients vs Eligible Patients Excluded Due To Incomplete Data eTable 2. Grid Search Results for Histogram Gradient Boosting Models eFigure. Prehospital Care Timelines for 3 Patients in the Included Cohort (n = 2809) eReference [file jamanetwopen-e259639-s001.pdf]

Supplementary Online Content

Weidman AC, Malakouti S, Salcido DD, et al. A machine learning trauma triage model for critical care transport. *JAMA Netw Open.* 2025;8(6):e259639. doi:10.1001/jamanetworkopen.2025.9639

eAppendix. SMOTE Analyses for Very Rare LSI
eTable 1. Demographic Profile of Included Patients vs Eligible Patients Excluded Due To Incomplete Data
eTable 2. Grid Search Results for Histogram Gradient Boosting Models
eFigure. Prehospital Care Timelines for 3 Patients in the Included Cohort (n = 2809)
eReference

This supplementary material has been provided by the authors to give readers additional information about their work.

© 2025 Weidman AC et al. *JAMA Network Open*.

eAppendix. SMOTE Analyses for Very Rare LSI

Several LSI subcategories were very rare, which can hamper model performance. To address this issue, for each LSI subcategory with a per-epoch rate less than 1%, we re-ran our ML model while setting the per-epoch positive rate to 3.0% via Synthetic Minority Oversampling Technique (SMOTE; (1)). In binary classification such as LSI/No LSI, the less frequent outcome class is considered the minority. Here, two-minute epochs that precede and LSI constitute the minority class, given that they are extremely rare across all LSI categories. SMOTE creates new ‘synthetic’ cases of the minority class training set by deriving feature values (e.g., physiologic and waveform statistics) that fall in the same feature space of, but do not replicate, existing training set minority cases. SMOTE continues to create synthetic cases until the minority class is represented at a specified rate. The 3.0% rate we chose was equivalent to the positive rate for airway intervention, the most frequent LSI category. We used feature-wise median imputation for these analyses, given that SMOTE requires complete data to synthesize new minority cases.

For thoracic and cardiovascular interventions, model performance was descriptively better when using SMOTE than in our original analyses and AUROC confidence intervals did not cross .50 (thoracic: AUROC = .724 [.514, .897]; cardiovascular: AUROC = .832 [.587, .997]). These findings suggest that our model performed above-chance when predicting cardiovascular and thoracic LSI, though still lower than for overall LSI. Modeling results using SMOTE were equivalent to the original analyses for blood transfusion and vasopressor medications (blood: AUROC = .744 [.675, .818]; vasopressors: AUROC = .838 [.758, .926]).

eTable 1. Demographic Profile of Included Patients vs Eligible Patients Excluded Due To Incomplete Data

		Included patients<br>*n* = 2,809	Eligible but excluded due to incomplete data<br>*n* = 1,197
Age (Years)	Mean	47.7	47.3
	SD	19.5	20.5
	Median	47.6	46.0
Gender	Male	1,981 (70.5%)	612 (67.8%)
	Female	775 (27.6%)	360 (30.1%)
	Other	53 (1.9%)	25 (2.1%)
Race	White	2,545 (90.6%)	1,073 (89.6%)
	Black	115 (4.1%)	58 (4.9%)
	Hispanic	55 (2.0%)	22 (1.8%)
	Other	12 (0.4%)	12 (1.0%)
	Unknown	12 (0.4%)	0 (0.0%)
	Missing	70 (2.5%)	32 (2.7%)
Weight (kg)	Mean	89.7	89.8
	SD	22.7	23.6
	Median	90.0	90.0

© 2025 Weidman AC et al. *JAMA Network Open*.

eTable 2. Grid Search Results for Histogram Gradient Boosting Models

**Hyperparameter**			
**Maximum Iterations**	**Maximum Tree Depth**	**Learning Rate**	**AUROC**
10	1	0.01	0.691
10	1	0.10	0.776
10	1	1.00	0.784
10	3	0.01	0.792
10	3	0.10	0.800
10	3	1.00	0.695
10	7	0.01	0.788
10	7	0.10	0.805
10	7	1.00	0.597
100	1	0.01	0.783
100	1	0.10	0.808
100	1	1.00	0.779
100	3	0.01	0.809
**100**	**3**	**0.10**	**0.813**
100	3	1.00	0.697
100	7	0.01	0.808
100	7	0.10	0.806
100	7	1.00	0.635
300	1	0.01	0.803
300	1	0.10	0.809
300	1	1.00	0.783
300	3	0.01	0.815
300	3	0.10	0.810
300	3	1.00	0.719
300	7	0.01	0.811
300	7	0.10	0.801
300	7	1.00	0.643

Note: Each row shows histogram gradient boosting (HGB) model performance under a different set of hyperparameter values (computed via five-fold cross-validation, as described in the manuscript)
Optimal hyperparameter combination is **bolded**
AUROC: Area under the receiver-operator curve

© 2025 Weidman AC et al. *JAMA Network Open*.

eFigure. Prehospital Care Timelines for 3 Patients in the Included Cohort (n = 2809)

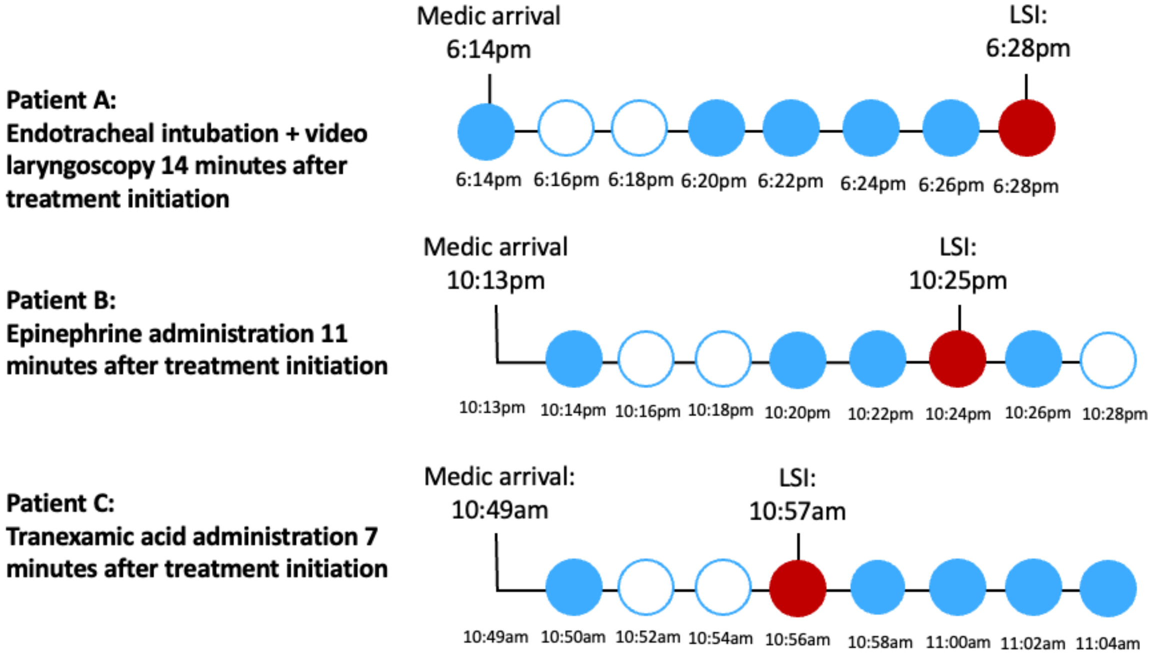

Note:
Solid circles represent two-minute epochs with recorded physiologic and waveform features
Empty circles represent two-minute epochs with missing features
Red circles: LSI occurred
Blue circles: LSI did not occur

© 2025 Weidman AC et al. *JAMA Network Open*.

eReference

1. Chawla NV, Bowyer KW, Hall LO, Kegelmeyer WP. SMOTE: synthetic minority over-sampling technique. Journal of artificial intelligence research. 2002 Jun 1;16:321-57.

© 2025 Weidman AC et al. *JAMA Network Open*.
