# Supplementary material for: A Machine Learning Trauma Triage Model for Critical Care Transport
Source: JAMA Netw Open. 2025 Jun 9;8(6):e259639. doi: 10.1001/jamanetworkopen.2025.9639 (PMC12150194; doi:10.1001/jamanetworkopen.2025.9639)
Supplement: Supplement 2. — Data Sharing Statement [file jamanetwopen-e259639-s002.pdf]

Data Sharing Statement

Weidman. A Machine Learning Trauma Triage Model for Critical Care Transport. *JAMA Netw Open*. Published June 09, 2025. doi:10.1001/jamanetworkopen.2025.9639

Data
Data available: No

Additional Information
Explanation for why data not available: Analytic code is available on the Open Science Framework (OSF; https://osf.io/4m6fn/); data is not publicly available due to our lab's ongoing research.
